# MHC polymorphism and disease resistance to *vibrio anguillarum *in 8 families of half-smooth tongue sole (*Cynoglossus semilaevis*)

**DOI:** 10.1186/1471-2156-12-78

**Published:** 2011-09-02

**Authors:** Min Du, Song-lin Chen, Yan-hong Liu, Yang Liu, Jing-feng Yang

**Affiliations:** 1 Key Lab for Sustainable Utilization of Marine Fisheries Resources, Ministry of Agriculture, Yellow Sea Fisheries Research Institute, Chinese Academy of Fishery Sciences, 266071, Qingdao, China; 2 College of Aqua-life Science and technology, Shanghai Ocean University, Shanghai 200090, China; 3 Honghe University, Mengzi, Yunnan Province,661100, China

**Keywords:** *Cynoglossus semilaevis*, *Vibrio anguillarum*, polymorphism, MHC IIB, susceptibility, resistance

## Abstract

**Background:**

Genes in the major histocompatibility complex (MHC) have a critical role in both the innate and adaptive immune responses because of their involvement in presenting foreign peptides to T cells. However, the nature has remained largely unknown.

**Results:**

We examined the genetic variation in MHC class IIB in half-smooth tongue sole (*Cynoglossus semilaevis*) after challenge with *vibrio anguillarum*. Two thousand and four hundred fry from 12 half-smooth tongue sole families were challenged with *Vibrio anguillarum*. To determine any association between alleles and resistance or susceptibility to *V. anguillarum*, 160 individuals from four high-resistance (HR, < 40.55% mortality) families and four low-resistance (LR, > 73.27% mortality) families were selected for MHC IIB exon2 gene sequence analysis. The MHC IIB exon2 genes of tongue sole displayed a high level of polymorphism and were discovered at least four loci. Meanwhile, the d_N_/d_S _[the ratio of non-synonymous (d_N_) substitutions to synonymous (d_S_) substitutions] in the peptide-binding region (PBR) was higher than that in the non-peptide-binding region (non-PBR). Eighty-eight alleles were discovered among 160 individuals, and 13 out of 88 alleles were used to analyze the distribution pattern between the resistant and susceptible families. Certain alleles presented in HR and LR with a different frequency, while other alleles were discovered in only the HR or LR families, not both. Five alleles, Cyse-DBB*6501, Cyse-DBB*4002, Cyse-DBB*6102, Cyse-DBB*5601 and Cyse-DBB*2801, were found to be associated with susceptibility to *V. anguillarum *with a frequency of 1.25%, 1.25%, 1.25%, 1.25% and 2.5% in the HR families, and 35%, 33.75%, 27.5%, 16.25%, 15% in the LR families (*p *< 0.01, 0.01, 0.01, 0.01, 0.01), respectively. Four alleles, Cyse-DBB*3301, Cyse-DBB*4701, Cyse-DBB*6801 and Cyse-DBB*5901, were found to be associated with resistance to *V. anguillarum*, with a frequency of 13.75%, 11.25%, 11.25%, 8.75% in the HR families and 1.25%, 1.25%, 1.25%, 1.25% and 1.25% in the LR families (*p *< 0.01, 0.05, 0.05 and p = 0.064), respectively.

**Conclusions:**

Elucidation of the role of MHC II B genes in half-smooth tongue sole should prove to be helpful to the in-depth development of marker-assisted selective breeding in half-smooth tongue sole.

## Background

Major histocompatibility complex (MHC) molecules play a critical role in both innate and adaptive immunity by presenting foreign peptides to T cells in vertebrate organisms, and have been considered candidate molecular markers of an association between polymorphisms and resistance/susceptibility to diseases [1]. A combination of balanced and directional selection is thought to be responsible for allelic variation of MHC genes in vertebrate populations, because pathogen pressure varies at different times and locations [2]. Two classes of MHC are found in fish, MHC class I and class II molecules. The genes encode glycoproteins which bind peptides for the presentation of self and non-self peptides to T-cell receptors (TCR) [3].

The MHC class II molecules are symmetrical heterodimers, consisting of one alpha chain and one beta chain, with non-covalent contacts in which the alpha1 and beta1 domains form a peptide-binding region (PBR). In mammals, MHC class II genes are constitutively expressed in antigen-presenting cells such as macrophages, B cells, monocytes and dendritic cells, and have direct functional relevance in the immune response. Class I antigens are expressed in all somatic cells [1,4,5]. In teleosts, class I and class II genes were found to reside on different linkage groups [6-8]. Many MHC genes have been isolated, characterized expressed and analyzed in at least 30 different fish species over the last twenty years [9-14]. Multiple loci and a considerable number of alleles at each given locus were found in the classical MHC genes. The peptide-binding region (PBR) contains the highest level of polymorphisms in the MHC genes [15-29]. Certain MHC alleles of the class II genes linked to viral and bacterial diseases have been reported in some species [30-37]. The link between disease susceptibility/resistance and MHC polymorphism is crucial for detecting MHC alleles related to resistance in marine aquaculture species for molecular marker-assisted selective breeding programs [38].

Half-smooth tongue sole (*Cynoglossus semilaevis*) is widely cultured throughout the coastal areas of North China [39]. However, viral and bacterial diseases frequently occur in this cultured fish, and losses due to infectious disease limit the profitability and the extent of the development of the aquaculture [40,41]. One pathogen which is a significant threat to half-smooth tongue sole is *Vibrio anguillarum *[42]. Antibiotics have partially solved problem, but antibiotic residues in fish, environmental pollution and antibiotic resistance are questions about which grave concerns remain [43]. Therefore, the selective breeding of tongue sole with disease resistance, basing on molecular techniques which can enhance the resistance to specific pathogens, may be a good approach to solving these problems.

The half-smooth tongue sole MHC class IIB cDNA sequence and cDNA polymorphisms have been reported [40]. However, the polymorphisms at the DNA level and the link between specific alleles and resistance to *V. anguillarum *have not been elucidated yet. In the present study, we investigated the single nucleotide polymorphism (SNP) sites and polymorphisms in MHC II B exon2, and the association between certain alleles and disease resistance or susceptibility to *Vibrio anguillarum*, across 8 families of half-smooth tongue sole.

## Methods

### Fish and rearing

Eighteen full-sib families were established as reported [44], using a method for producing strains with a high growth rate and disease resistance. Male parents came from wild populations while female parents came from farming populations. Fertilized ova were hatched and reared at the breeding station at Minbo aquatic Co., Ltd. Located in Laizhou city, Shandong province, China. Each family was kept in a separate tank. The fry were fed a commercial diet using a standard feeding regimen [45].

### Challenge test

For the challenge test, 200 individuals of each family (12 out of 18 families were large enough to be included), ten months old, were intraperitoneally injected with a 0.2 ml bacterial suspension of approximately 10,000,000 cells of *V. anguillarum*, while 16 individuals were injected with 0.9% saline as control [15]. Each fry weighed approximately 12-15 grams. The fry of each family were kept in a 1 m^3 ^single tank with a fresh seawater supply at 23°C. This challenge experiment was performed twice and lasted for approximately two weeks. Mortality was recorded every day and the fin clips of all the fish were collected and preserved in absolute ethanol until use. The gross signs of fish mortality were based on a previous reporting method [42].

### Sampling and DNA isolation

To identify whether MHC IIB exon2 alleles are associated with resistance or susceptibility to *V. anguillarum*, fin samples from each family of half-smooth tongue sole were collected and recorded from the first 20 to die and the last survivors at the time the bacterial challenge was terminated and preserved in absolute ethanol until use. High-resistance families (HR) with a survival rate (SR) > 59.45% and susceptible families or low-resistance families (LR) with a SR < 26.73% were selected from the challenge trials. The numbers fish which died or survived after the infection recorded for each family (Additional file 1).

Genomic DNA was isolated from the dorsal or caudal fin samples of 20 individuals per family (from the 4LR and 4HR families) using the phenol-chloroform method as described by Chen *et al. *[46]. The quality and concentration of DNA were assessed by agarose gel electrophoresis and then measured with a GENEQUANT Pro (Pharmacia Biotech Ltd.) RNA/DNA spectrophotometer. Finally, DNA was adjusted to 100 ng/μl and stored at -20°C.

### Primer design and Polymerase Chain Reaction (PCR)

A pair of gene-specific primers was used for the PCR amplification of the MHC II B gene: hMPN12 (5'-CTCTCTTCTCTTCCTCCTCAC-3') and hMPC12 (5'-ACA CTCACCTGATTTAGCCA-3'). They were designed according to reported half-smooth tongue sole MHC II B cDNA sequences [40]. The primer pair was used to amplify part of exon1, and all of intron1 and exon2 from half-smooth tongue sole using a Polymerase Chain Reaction technique. A 25 μl PCR reaction mixture contained 1 μl of template DNA, 2.5 μl of 10×*Taq *polymerase buffer (TransGen Biotech), 1.5 mM MgCl_2_, 0.2 mM dNTP mix, 0.2 μM of the forward and reverse primers, and 1 unit of *Taq *polymerase (TransGen Biotech). The amplifications were performed on a Peltier Thermal Cycler (PTC-200). A Molecular Imager Gel Doc XR system (Bio-rad) was used to determine the PCR products by electrophoresis on a 1% agarose gel.

### Cloning and sequencing

The PCR products were resolved by electrophoresis on 1.5% agarose gels. The fragments of interest were excised and purified with the QIAEX II gel extraction kit (Qiagen). The purified fragments were cloned into a PBS-T vector (Takara) according to the standard PBS-T vector protocol (Takara) and then transformed into TOP 10 *Escherichia coli *competent cells (TransGen Biotech). Forward and reverse M13 primers were used to screen for positive clones via PCR. Ten positive clones from the upper purified fragments were sequenced with an ABI 3730 automated sequencer using the M13+/- primer.

### Genotyping, sequence analysis and statistical tests analysis

Sequence data were analyzed using DNASTAR 5.0 and DNAMAN software. The alignment was performed with MEGA4.0 [47]. The rate of synonymous substitution (d_S_) and non-synonymous substitution (d_N_) was calculated accord with an earlier report [47] using MEGA4.0 software. DAMBE and DnaSP5.0 software packages were used to analyze the polymorphisms [48]. Statistical analysis was carried out with SPSS13.0. Differences in the allelic frequency were verified using Fisher's exact test and the significance level [49] was determined for every individual (n = 160) and each family (n = 8).

The new alleles were designated Cyse-DBB*0101 to Cyse-DBB*6601 on the basis of the rules reported by Davies *et al. *[50]. *Cyse *refers to *Cynoglossus semilaevis*, D to class II, the first B to an uncharacterized family and the second B to β chain-encoding genes. In the first four digits after the asterisk, the first two digits refer to the major type (alleles that differ by at least five amino acid substitutions), while the last two digits refer to the subtype (alleles that differ by less than five amino acid substitutions within a single major type) [51,52].

## Results

### To analyze disease resistance among 12 half-smooth tongue sole families

The first specific mortality appeared after 16 h due to an ip injection of *V. anguillarum*, and the challenge test lasted two weeks, at which time the overall accumulated mortality reached 42.24%. The survival rate among the 12 test families ranged from 15% to79.25%, which was determined on the basis of each family. Here, we selected four high-resistance and four low-resistance families to ascertain whether MHC IIB exon2 alleles were associated with resistance to *V. anguillarum *among the 12 families of half-smooth tongue sole. The mean prevalence of survival of the four high-resistance families was 59.45%, while that of the four low-resistance families was considerably less at 26.73%.

### To elucidate sequence polymorphism within exon2 of MHC IIB gene in 8 half-smooth tongue sole families

Eighty individuals from the four high-resistance families and eighty individuals from the four low-resistance families were used in the present study (Additional file 1). Nine to twelve positive clones per individual were sequenced and 1618 sequences were obtained. A fragment of 397 bp was obtained in reference to the complete half-smooth tongue sole MHC IIB cDNA sequence [40] and intron-exon boundary GT-AG rule. This fragment of 397 bp contains a part of exon1 (35 bp), the entire intron1 (84 bp, containing a 12 bp CA repeat sequence) and the entire exon2 of MHC IIB. A fragment of 270 bp containing the complete exon2 which encodes the β1 domain of the MHC IIB gene was also analyzed. The results indicated 88 different sequences, in which 88 novel alleles were designated (Table 1) belonging to 57 major allele types, following established allele nomenclature method [49,50].

**Table 1:** Alleles and Genbank Accession Number of half-smooth tongue sole MHC class II exon2 gene

Allele	GenBank Accession **No**.	Allele	GenBank Accession **No**.	Allele	GenBank Accession **No**.
Cyse-DBB*0101	GU194838	Cyse-DBB*2401	GU194876	Cyse-DBB*4601	GU194918
Cyse-DBB*0102	GU194839	Cyse-DBB*2501	GU194877	Cyse-DBB*4602	GU194919
Cyse-DBB*0201	GU194840	Cyse-DBB*2601	GU194878	Cyse-DBB*4701	GU194921
Cyse-DBB*0202	GU194841	Cyse-DBB*2602	GU194879	Cyse-DBB*4801	GU194922
Cyse-DBB*0301	GU194842	Cyse-DBB*2603	GU194880	Cyse-DBB*4802	GU194923
Cyse-DBB*0401	GU194843	Cyse-DBB*2801	GU194882	Cyse-DBB*4803	GU194924
Cyse-DBB*0701	GU194847	Cyse-DBB*2802	GU194883	Cyse-DBB*5002	GU194927
Cyse-DBB*0801	GU194848	Cyse-DBB*2803	GU194884	Cyse-DBB*5003	GU194928
Cyse-DBB*0901	GU194850	Cyse-DBB*2901	GU194886	Cyse-DBB*5101	GU194929
Cyse-DBB*1001	GU194851	Cyse-DBB*3002	GU194888	Cyse-DBB*5202	GU194932
Cyse-DBB*1002	GU194852	Cyse-DBB*3101	GU194889	Cyse-DBB*5401	GU194934
Cyse-DBB*1003	GU194853	Cyse-DBB*3102	GU194890	Cyse-DBB*5501	GU194935
Cyse-DBB*1201	GU194855	Cyse-DBB*3201	GU194891	Cyse-DBB*5601	GU194936
Cyse-DBB*1301	GU194856	Cyse-DBB*3301	GU194892	Cyse-DBB*5602	GU194937
Cyse-DBB*1402	GU194858	Cyse-DBB*3302	GU194893	Cyse-DBB*5604	GU194939
Cyse-DBB*1403	GU194859	Cyse-DBB*3401	GU194896	Cyse-DBB*5701	GU194940
Cyse-DBB*1501	GU194860	Cyse-DBB*3501	GU194897	Cyse-DBB*5801	GU194941
Cyse-DBB*1601	GU194861	Cyse-DBB*3701	GU194902	Cyse-DBB*5901	GU194942
Cyse-DBB*1602	GU194862	Cyse-DBB*3702	GU194903	Cyse-DBB*5902	GU194943
Cyse-DBB*1701	GU194864	Cyse-DBB*3901	GU194905	Cyse-DBB*6001	GU194944
Cyse-DBB*1702	GU194865	Cyse-DBB*4001	GU194906	Cyse-DBB*6002	GU194945
Cyse-DBB*1703	GU194866	Cyse-DBB*4002	GU194907	Cyse-DBB*6102	GU194947
Cyse-DBB*1801	GU194867	Cyse-DBB*4101	GU194910	Cyse-DBB*6201	GU194948
Cyse-DBB*2002	GU194870	Cyse-DBB*4201	GU194911	Cyse-DBB*6301	GU194949
Cyse-DBB*2101	GU194871	Cyse-DBB*4301	GU194912	Cyse-DBB*6401	GU194950
Cyse-DBB*2201	GU194872	Cyse-DBB*4302	GU194913	Cyse-DBB*6402	GU194951
Cyse-DBB*2202	GU194873	Cyse-DBB*4402	GU194915	Cyse-DBB*6403	GU194952
Cyse-DBB*2203	GU194874	Cyse-DBB*4501	GU194916	Cyse-DBB*6404	GU194954
Cyse-DBB*2301	GU194875	Cyse-DBB*4502	GU194917	Cyse-DBB*6501	GU194955
				Cyse-DBB*6601	GU194956

Gaps were not found in the full alignment of the 270 bp exon2 of the MHC IIB gene. A putative 90 amino acid peptide was based on a sequence alignment with the half-smooth tongue sole MHC II B cDNA sequence [40]. Among the 270 nucleotides, 72 regions and 121(44.8%) nucleotide positions were variable. The numbers of two-nucleotide mutation, three-nucleotide mutation and four-nucleotide mutation were 24, 11 and 1, respectively (Table 2). At the SNP sites, there were two kinds of nucleotide substitutions, i. e. transition (Table 2, Serial No. 1, 7, 11, 13, 18, 23, 28, 29, 32, 33, 35, 42, 43, 44, 46, 49, 52, 53, 54, 60 and 69) and transversion (Table 2, Serial No. 20, 21, 25, 59). Three kinds of mutation per site (Table 2, Serial No. 2, 4, 6, 9, 14, 15, 16, 22, 26, 30, 31, 36, 37, 41, 51, 56, 58, 61, 62, 63, 65, 67, 68 and 71) which revealed the mutation hotspots. 36 out of 72 mutation regions were multi-nucleotide co-mutations, ranging from two to five nucleotides per region. The SNP sites were located in a tight region from position 9 to 29 (Table 2), so this were most of the mutation hotspots of MHC exon2 herein must be located. The frequency ratio ranged from 0.989:0.011 (Table 2, Serial No.1, 23, 32, 49, 59 and 60) to 0.557:0.443 (Table 2, Serial No.7). No frame-shift mutation was observed in these sequences. The peptide binding regions in half-smooth tongue sole MHC II B were based on the corresponding peptide binding region identified in humans [53].

**Table 2:** Distribution of SNP sites within exon2 of MHC IIB allelic sequences of half-smooth tongue sole

Serial number	Position	Base type	**Allele no**. (n = 88)	Frequency	Serial number	Position	Base type	Allele no. (n = 88)	Frequency
1	6	T	87	0.989	39	104-106	ATC	51	0.580
		C	1	0.011			ATT	1	0.011
2	9-11	CTA	52	0.591			ATG	1	0.011
		GTA	1	0.011			CAG	35	0.398
		GAG	35	0.398	40	109-111	TCG	84	0.955
3	12	C	17	0.193			TCA	2	0.023
		T	34	0.386			CCG	1	0.011
		A	7	0.080			TTG	1	0.011
		G	30	0.341	41	124-126	GGA	49	0.557
4	13	A	82	0.932			AGA	12	0.136
		T	5	0.057			GAG	27	0.307
		G	1	0.011	42	130	A	56	0.636
5	14-15	AT	30	0.341			T	32	0.364
		AC	1	0.011	43	143	C	2	0.023
		TT	55	0.625			T	86	0.977
		CT	2	0.023	44	148-149	AT	49	0.557
6	16	C	32	0.364			TA	8	0.091
		T	20	0.227			TT	31	0.352
		A	36	0.409	45	156-157	CC	1	0.011
7	18	G	39	0.443			TA	24	0.273
		A	49	0.557			TC	63	0.716
8	19	T	33	0.375	46	163	C	87	0.989
		G	20	0.227			T	1	0.011
		C	30	0.341	47	168-169	AG	71	0.807
		A	5	0.057			GC	17	0.193
9	20	G	46	0.523	48	170-172	ATG	75	0.852
		A	34	0.386			ATT	9	0.102
		C	8	0.091			TTG	3	0.034
10	21-23	ACA	53	0.602			ACT	1	0.011
		GTG	35	0.398	49	174	G	1	0.011
11	24	G	74	0.841			A	87	0.989
		A	14	0.159	50	177-178	GA	86	0.977
12	25	A	35	0.398			AA	1	0.011
		G	31	0.352			GG	1	0.011
		T	19	0.216	51	181-183	GTC	80	0.909
		C	3	0.034			ATC	7	0.080
13	26	T	5	0.057			GAA	1	0.011
		C	83	0.943	52	193	C	24	0.273
14	28-29	CC	52	0.591			T	64	0.727
		CA	1	0.011	53	196	A	62	0.705
		GA	35	0.398			G	26	0.295
15	32-33	CG	35	0.398	54	198	A	69	0.784
		TG	1	0.011			G	19	0.216
		TC	52	0.591	55	199-200	GG	40	0.455
16	38-39	CA	52	0.591			GT	18	0.205
		CG	1	0.011			TG	8	0.091
		TA	35	0.398			CG	22	0.25
17	40-41	AC	51	0.580	56	205	A	67	0.761
		GC	1	0.011			C	20	0.227
		AT	35	0.398			G	1	0.011
		CT	1	0.011	57	207-208	GA	62	0.705
18	44	C	36	0.409			AC	3	0.034
		T	52	0.591			GG	8	0.091
19	47-49	AAA	52	0.591			GC	5	0.057
		AAG	1	0.011	58	210-211	AA	86	0.977
		TAA	19	0.216			GA	1	0.011
		TGA	16	0.182			AT	1	0.011
20	51	G	53	0.602	59	218	G	87	0.989
		C	35	0.398			T	1	0.011
21	53	G	36	0.409	60	220	A	87	0.989
		C	52	0.591			G	1	0.011
22	55-56	AC	69	0.784	61	226-227	TG	47	0.534
		AT	18	0.205			TA	40	0.455
		GC	1	0.011			CG	1	0.011
23	58	A	87	0.989	62	228	A	50	0.568
		G	1	0.011			C	29	0.330
24	63-64	GC	51	0.580			T	9	0.102
		GT	1	0.011	63	229	A	82	0.932
		GA	1	0.011			T	2	0.023
		CA	35	0.398			G	4	0.045
25	67	A	68	0.773	64	231-234	AAC	61	0.693
		T	20	0.227			ACT	2	0.022
26	72	C	13	0.148			CAC	20	0.227
		G	40	0.455			AGC	5	0.057
		T	35	0.398	65	237-238	GG	79	0.898
27	74	C	40	0.455			GA	5	0.057
		G	48	0.545			AG	4	0.045
28	78	C	38	0.432	66	240-241	AA	10	0.114
		T	50	0.568			AT	54	0.614
29	80	C	35	0.398			CT	23	0.261
		T	53	0.602			GT	1	0.011
30	82-83	AC	52	0.591	67	242-243	TG	46	0.523
		AT	35	0.398			TT	19	0.216
		GT	1	0.011			GG	23	0.261
31	84-85	TT	30	0.341	68	245-246	CT	68	0.773
		CT	23	0.261			CA	19	0.216
		TA	35	0.398			AT	1	0.011
32	87	A	87	0.989	69	248	T	2	0.023
		G	1	0.011			C	86	0.977
33	90	A	86	0.977	70	250-253	ACCA	10	0.114
		G	2	0.023			ACGC	64	0.727
34	92-94	ACT	52	0.591			AGCC	1	0.011
		GAG	36	0.409			GGAC	12	0.136
35	96	G	35	0.398			ACGG	1	0.011
		A	53	0.602	71	254-256	TGC	70	0.796
36	98-99	GA	42	0.477			TGG	12	0.136
		GT	11	0.125			GTT	6	0.068
		AT	35	0.398	72	256-258	TT	12	0.136
37	100	C	1	0.011			TC	63	0.716
		A	38	0.432			CC	2	0.023
		T	49	0.557			TG	11	0.125
38	101-102	CA	34	0.386					
		CG	16	0.182					
		GA	3	0.034					
		TG	31	0.352					
		TA	4	0.046					

The variable positions of the PBR comprised 20 (87%) out of 23 and the polymorphic nucleotide PBR sites were 40 (57.97%) of 69. In the putative peptide-binding region, the ratio of non-synonymous (d_N_) substitution (0.261) was 1.7 times higher than that of synonymous (d_S_) substitution (0.153). The rates of d_N _and d_S _in the non-PBR were 0.087 and 0.159, respectively. All of the sequences were used to calculate these rates. The rate of d_s _in the non-PBR(0.159) was slightly higher than that of d_S _in the PBR(0.153), and d_N _in the PBR (0.261) occurred at a significantly higher rate than that in the non-PBR (0.087), but d_S _in the PBR (0.153) was a little lower than that in the non-PBR (0.159) (Table 3).

**Table 3:** Synonymous (dS) and nonsynonymous (dN) substitution rate in the putative peptides binding region (PBR) and non-peptides binding region (non-PBR) among half-smooth tongue sole alleles

Region	No. of codons	**d**_**N**_**(SE)**	d_S_(SE)	**d**_**N**_**/d**_**S**_
PBR	23	0.261 ± 0.033	0.153 ± 0.052	1.70
Non-PBR	67	0.087 ± 0.016	0.159 ± 0.034	0.547
Total	90	0.132 ± 0.017	0.157 ± 0.027	0.841

**Figure 1:**
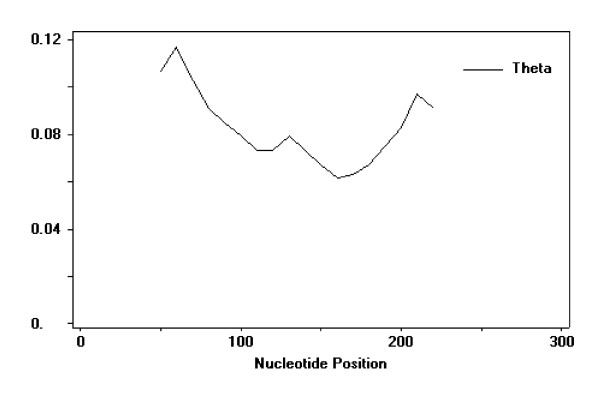
**The nucleotide diversity within exon2 sequences of MHC IIB genes at the 88 alleles denoted by Theta-W**. Sliding window length: 100; step size: 10.

The per site nucleotide diversity Pi (p) was 0.13785, and per the site Theta-W value of the 88 sequences was 0.08876. Ninety-six out of the 121 variable sites were parsimony informative sites. The haplotype diversity (H) and the average number of nucleotide differences (k) were 1 and 37.220, respectively. DnaSP5.0 software was used to calculate these polymorphic values. The exon2 sequence of MHC IIB indicated high nucleotide diversity in the 8 families of tongue sole. Figure 1 shows the spatial distribution of the nucleotide diversity. Two peaks appeared at the downstream and upstream of exon2 of the MHC IIB sequences, respectively, while the Theta-W value in the middle region was lower.

### To identify association between the MHC IIB alleles and disease resistance/susceptibility to *V. anguillarum *in half-smooth tongue sole

**Figure 2:**
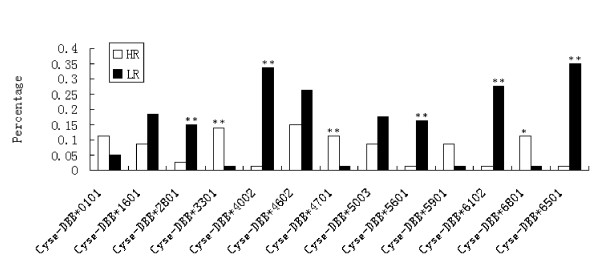
**Sequence polymorphism analysis within exon 2 of MHCIIB gene**. (Asterisks indicate the correlative amino acid that combines the antigen).

**Figure 3:**
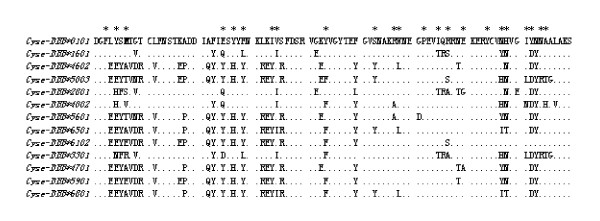
**Distribution of MHC class IIB alleles in high-resistance families individuals (white bars) and low-resistance families individuals (black bars) of half-smooth tongue sole**. *Asterisks denote P < 0.05. ** denote P < 0.01.

Additional file 2 shows the number of alleles per individual and the comparative individual number. An average ten clones per individual were sequenced, and 2 to 7 alleles per individual were discovered, which inferred the existence of at least seven alleles and four loci of the MHC IIB gene, in accordance with the reports of Xu *et al. *[40]. Among the 8 families examined, only 2.5% of the individuals were homozygous (all families were heterozygous) for exon2 of the MHC class IIB gene of tongue sole. Eighty-eight sequences resulted in eighty-eight different MHC IIB exon2 alleles deduced from 160 individuals. The distribution of the alleles was unequal. Certain alleles had a low frequency and were excluded from allele distribution analysis between the HR and LR families. Thirteen alleles were used for distribution analysis (Figure 2). The alleles Cyse-DBB*3301, Cyse-DBB*4701, Cyse-DBB*6801 and Cyse-DBB*5901 were more prevalent in individuals from the HR families (P = 0.005, 0.018, 0.018 and 0.064, respectively n = 160 individuals), while Cyse-DBB*6501, Cyse-DBB*4002, Cyse-DBB*6102, Cyse-DBB*5601 and Cyse-DBB*2801 were more prevalent in individuals from low-resistance families, as shown by chi-square test (P < 0.01, 0.01, 0.01, 0.01, 0.01 respectively n = 160 individuals). Some alleles were not significantly different in the HR and LR families, such as Cyse-DBB*0101(P = 0.247), Cyse-DBB*1601(P = 0.107), Cyse-DBB*4602 (P = 0.117) and Cyse-DBB*5003 alleles (P = 0.159). Here we (deduced) show that Cyse-DBB*3301, Cyse-DBB*4701, Cyse-DBB*6801 and Cyse-DBB*5901 were associated with resistance, while Cyse-DBB*6501, Cyse-DBB*4002, Cyse-DBB*6102, Cyse-DBB*5601 and Cyse-DBB*2801 were associated with susceptibility to *V. anguillarum *in half-smooth tongue sole. Alignment of the 13 deduced MHC IIB amino acid sequences (Figure 3) indicated that no specific single amino acid substitution was evidently involved in the resistance or susceptibility, as there was no specific amino acid substitution difference between the HR families and LR families.

## 4. Discussion

It is well known that MHC genes are vital components of both the innate and adaptive immune system. They present foreign peptides to T cells. Cloning and cDNA polymorphism of the MHC II B gene has been discussed [40]. In the present study, partial sequences of the MHC class IIB gene in different families of half-smooth tongue sole were isolated, then molecular polymorphisms as well as the link between alleles and resistance/susceptibility to *V. anguillarum *were analyzed.

Among the 72 mutated regions in the complete sequence of MHC IIB exon2, 36 regions were multi-nucleotide co-mutations, which indicate inter-allelic recombination took place in these regions. Moreover, no deletion, insertion or stop codon was observed, indicating that all of these alleles were functional genes. The frequency ratio of substituted nucleotides per mutation region was not equally distributed, which suggests that different regions might have different impact.

The rate of non-synonymous substitutions to synonymous substitutions (d_N_/d_S_) in the PBR and non-PBR of MHC IIB exon2 of half-smooth tongue sole was studied (Table 3). The d_N_/d_S _ratio was higher in the PBR than non-PBR, which corresponds with the results reported in other species [43,54-56]. The d_N_/d_S _ratio in exon2 was higher than 1. The location of the PBR sites in the MHC genes of fish was not yet defined, therefore PBR sites were identified using the model of Brown *et al. *[53] to define *HLA-DRB*, It was also in accordance with a previous application by Xu *et al. *[38] for half-smooth tongue sole. The 23 positions were used as PBR sites for in-depth study: 3, 5, 7, 25, 27, 29, 34, 35, 44, 53, 57, 58, 62, 65, 67, 71, 74, 77, 78, 82, 83, 85 and 86 (Figure 3).

It is possible that the PBR sites in fish do not exactly correspond to those in humans [57]. In mammals, MHC polymorphisms are maintained over long periods of time by balanced selection or positive selection at the non-synonymous sites specifying the PBR of the MHC [7]. The ratio between non-synonymous and synonymous substitutions in PBR sites of MHC IIB exon2 genes is greater than 1 (Table 3), as would be expected if the locus were evolving under a condition of balanced selection [58]. The number of alleles per individual ranged from 1 to 5, which showed that at least three loci existed per individual, a result is in accordance with previous studies [22,28,40]. Polymorphism of the 88 alleles in the 160 individuals was higher in half-smooth tongue sole than in Atlantic salmon [57,59] and cyprinid fish [54], and each family had 25-38 alleles. A few hypotheses have been put forward to interpret the abundant polymorphism of the MHC genes, including overdominant selection or heterozygous advantage [60], negative frequency-dependent selection [61,62] and balanced selection [24]. Pathogen-driven selection [26,60] is reported to be contributing to MHC gene diversity through both frequency-dependent selection and heterozygote advantage (over-dominance) [15]. In the present study, the high rate of d_N_/d_S _score and high levels of polymorphism which occurred in half-smooth tongue sole revealed that balanced selection is responsible for presence in the PBR domain of the MHC class IIB exon2 gene. This results in the high polymorphism levels in MHC IIB genes in half-smooth tongue sole. Due to the polymorphic nature of MHC genes, certain alleles/haplotypes may be associated with increased disease resistance. In the present study, the distinct distribution pattern of the alleles exhibited a relationship between MHC class IIB alleles and resistance/susceptibility to *V. anguillarum *in half-smooth tongue sole.

The Cyse-DBB*3301, Cyse-DBB*4701 and Cyse-DBB*6801 alleles which was found in three families, and the Cyse-DBB*5901 allele in two families, were markedly more frequent in HR families (13.75%, 11.25%, 11.25%, 8.75% respectively) than in LR families (1.25%, 1.25%, 1.25%, 1.25%, respectively). This suggests an association of the *V. anguillarum *disease resistance alleles in half-smooth tongue sole. The Cyse-DBB*6501, Cyse-DBB*4002 and Cyse-DBB*5601 alleles were found in two LR families (35%, 33.75% and 16.25% respectively) and one HR family (1.25%, 1.25% and 1.25%, respectively), while the Cyse-DBB*6102 allele was found in three LR families (27.5%) and one HR family (1.25%), Cyse-DBB*2801 was found in two LR families (15%) and two HR families (2.5%), which might be associated with susceptibility to *V. anguillarum *in half-smooth tongue sole. In the present study, statistical analysis was used to reveal the associations between the alleles and resistance or susceptibility to *V. anguillarum *in half-smooth tongue sole. The observed link between alleles Cyse-DBB*3301, Cyse-DBB*4701, Cyse-DBB*6801, Cyse-DBB*5901, Cyse-DBB*6501, Cyse-DBB*4002, Cyse-DBB*6102, Cyse-DBB*5601 and Cyse-DBB*2801 and resistance/susceptibility to *V. anguillarum *supported the hypothesis that frequency-dependent selection is crucial for the maintenance of MHC variation [63]. This experimental result was in accord with reports in Atlantic salmon [64] and flounder [38]. However, it was not possible to identify a single allele which appeared in all HR families or all LR families. This might indicate the importance of multiple polymorphisms. One MHC haplotype has been reported to be significantly associated with resistance to Marek's disease in chickens [65], and MHC polymorphism was significantly associated with both juvenile survival and resistance to nematode parasites was also reported in Soay sheep [31].

A link between MHC polymorphism and resistance/susceptibility to disease in fish has been reported. Kjøglum *et al. *[5] demonstrated that fish with the genotypes UBA*0201/UBA*030 and DAA*0201/*0201 were the most resistant to infectious anaemia in Atlantic salmon, while fish with the genotypes UBA*0601/*080, DAA*0501/*0501 and UBA*0201/*030, DAA*0301/*0501 were the most susceptible, based on an analysis of the combined MHC class I and class II A genotypes. It is reported [15] that the allele combinations DAA*0201-*0201 and DAA*0301-*0301 displayed a significantly lower prevalence of death in homozygous fish than in Atlantic salmon containing one copy or no copy of the allele in *Aeromonas salmonicida*-challenged Atlantic salmon.

The *Sasa-DAA-3'UTR 239 *allele [36] was shown to be significantly associated with a decrease in the severity of amoebic gill disease in Atlantic salmon. It was also reported [66] that *Sasa-B-04*, at the non-classical class I locus, was highly associated with resistance to infectious hematopoietic necrosis in Atlantic salmon. The alleles Paol-DAB*4301 and Paol-DAB*1601 were shown to be associated with resistance and susceptibility to *V. anguillarum *in flounder [38].

In this study in half-smooth tongue sole, the alleles Cyse-DBB*3301, Cyse-DBB*4701, Cyse-DBB*6801 and Cyse-DBB*5901 were found to be associated with resistance while the Cyse-DBB*6501, Cyse-DBB*4002, Cyse-DBB*6102, Cyse-DBB*5601 and Cyse-DBB*2801 alleles were associated with susceptibility to *V. anguillarum*. Associations of MHC with resistance or susceptibility to specific pathogens can also be derived through linkage disequilibrium with a resistance or susceptibility locus or gene, and may not be due to the MHC gene itself [55,67-69].

## Conclusions

It can not ruled out that another linked gene, individual genetic background and different strains or populations may to some extent have caused the observed link, but here the Cyse-DBB*3301, Cyse-DBB*4701, Cyse-DBB*6801 and Cyse-DBB*5901 alleles were associated with resistance to *V. anguillarum*, while the Cyse-DBB*6501, Cyse-DBB*4002, Cyse-DBB*6102, Cyse-DBB*5601 and Cyse-DBB*2801 alleles were associated with susceptibility to *V. anguillarum *in half-smooth tongue sole. Further studies are needed to confirm the association between MHC class IIB exon2 gene with resistance to *V. anguillarum *in half-smooth tongue sole.

## Authors' contributions

Professor SLC and MD designed of the study. MD carried out the molecular genetic studies, participated in the sequence alignment and wrote the final drafts of the manuscript. Professor SLC and YHL provided academic advising of this study. YL participated in the manuscript revision. MD and JFY were in charge of fish breeding. All authors read and approved the final manuscript.

## Supplementary Material

Additional file 1 **Results of the infection with bacterial**. Results of the infection with bacterial is presented. Numbers of high-resistance (HR, survivor rate(SR) > 59.45% when infected with the bacterium *Vibrio anguillarum*) and low-resistance (LR, SR < 26.73%)families of *Cynoglossus semilaevis *from which dead, surviving individuals were sampled. Click here for file

Additional file 2 **The individual ID and corresponding number of allele**. We presented the number of alleles per individual of half-smooth tongue sole and its corresponding individual number. Click here for file
