# Supplementary material for: MHC polymorphism and disease resistance to vibrio anguillarum in 8 families of half-smooth tongue sole (Cynoglossus semilaevis)
Source: BMC Genet. 2011 Sep 2;12:78. doi: 10.1186/1471-2156-12-78 (PMC3199252; doi:10.1186/1471-2156-12-78)
Supplement: Additional file 1 — Results of the infection with bacterial. Results of the infection with bacterial is presented. Numbers of high-resistance (HR, survivor rate(SR) > 59.45% when infected with the bacterium Vibrio anguillarum) and low-resistance (LR, SR < 26.73%)families of Cynoglossus semilaevis from which dead, surviving individuals were sampled. [file 1471-2156-12-78-S1.DOC]

| Family | Individuals per family | | Total |
| --- | --- | --- | --- |
|  | Dead | Surviving |  |
| HR 4  LR 4  Total 8 | 20  80 | 20  80 | 80  80  160 |
