# Supplementary material for: MHC polymorphism and disease resistance to vibrio anguillarum in 8 families of half-smooth tongue sole (Cynoglossus semilaevis)
Source: BMC Genet. 2011 Sep 2;12:78. doi: 10.1186/1471-2156-12-78 (PMC3199252; doi:10.1186/1471-2156-12-78)
Supplement: Additional file 2 — The individual ID and corresponding number of allele. We presented the number of alleles per individual of half-smooth tongue sole and its corresponding individual number. [file 1471-2156-12-78-S2.DOC]

| Individual no. 10 32 39 49 24 6  Allele no. 2 3 4 5 6 7 |
| --- |
